# Supplementary material for: Synergistic Responses of Tibetan Sheep Rumen Microbiota, Metabolites, and the Host to the Plateau Environment
Source: Int J Mol Sci. 2023 Oct 3;24(19):14856. doi: 10.3390/ijms241914856 (PMC10573510; doi:10.3390/ijms241914856)
Supplement: Supplementary file 1 [file ijms-24-14856-s001.zip › Fig S1-S9,Table S1.docx]

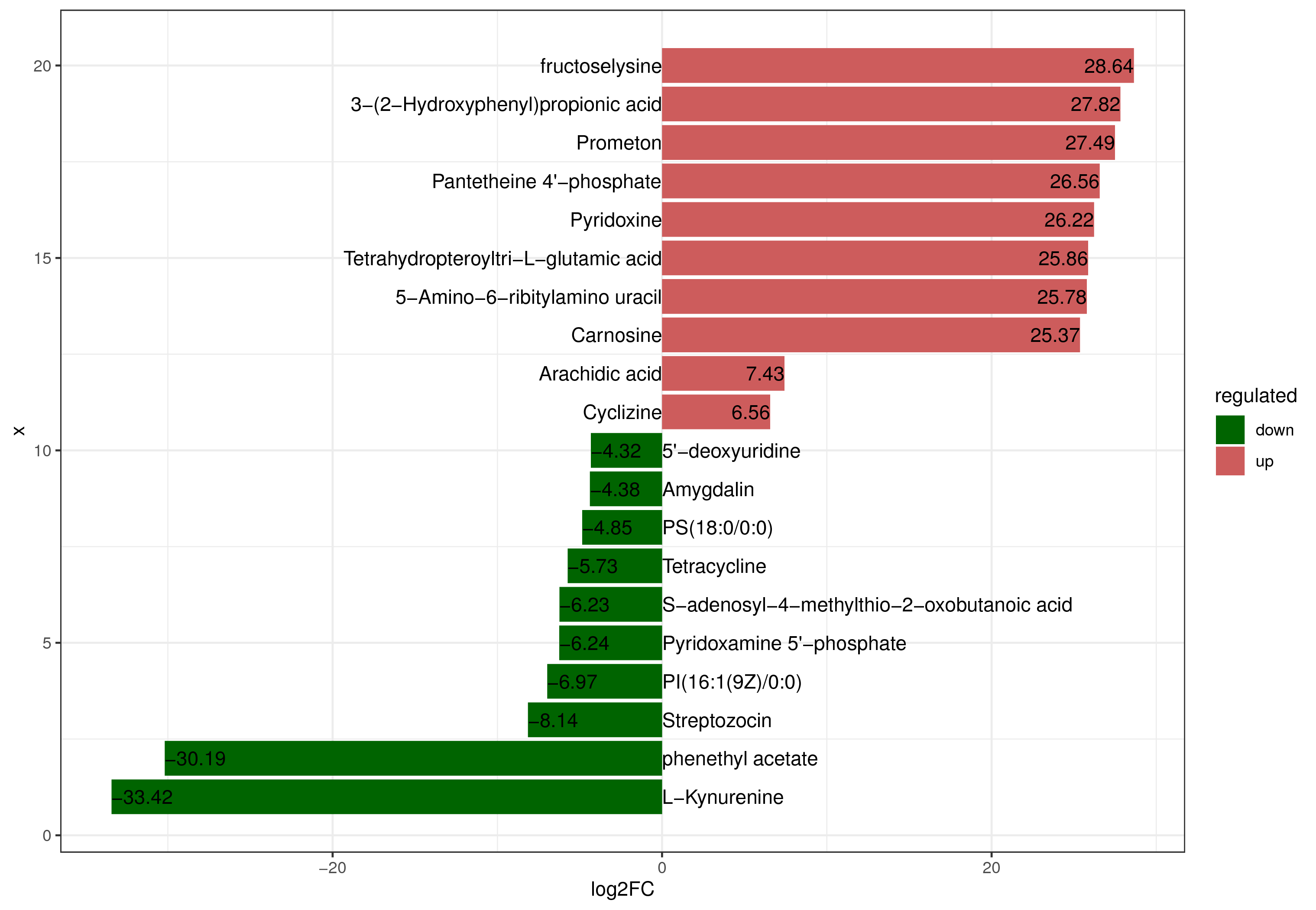

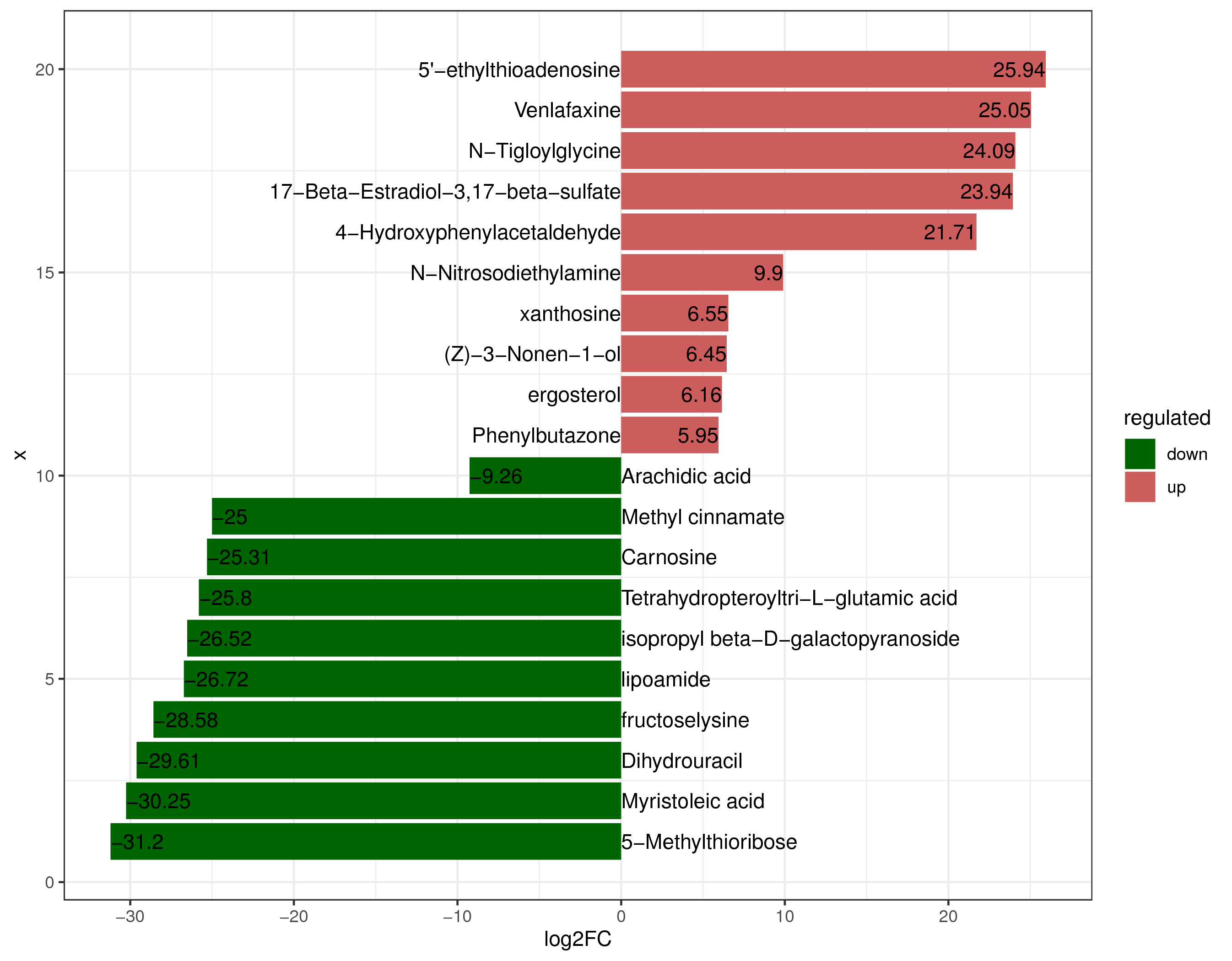

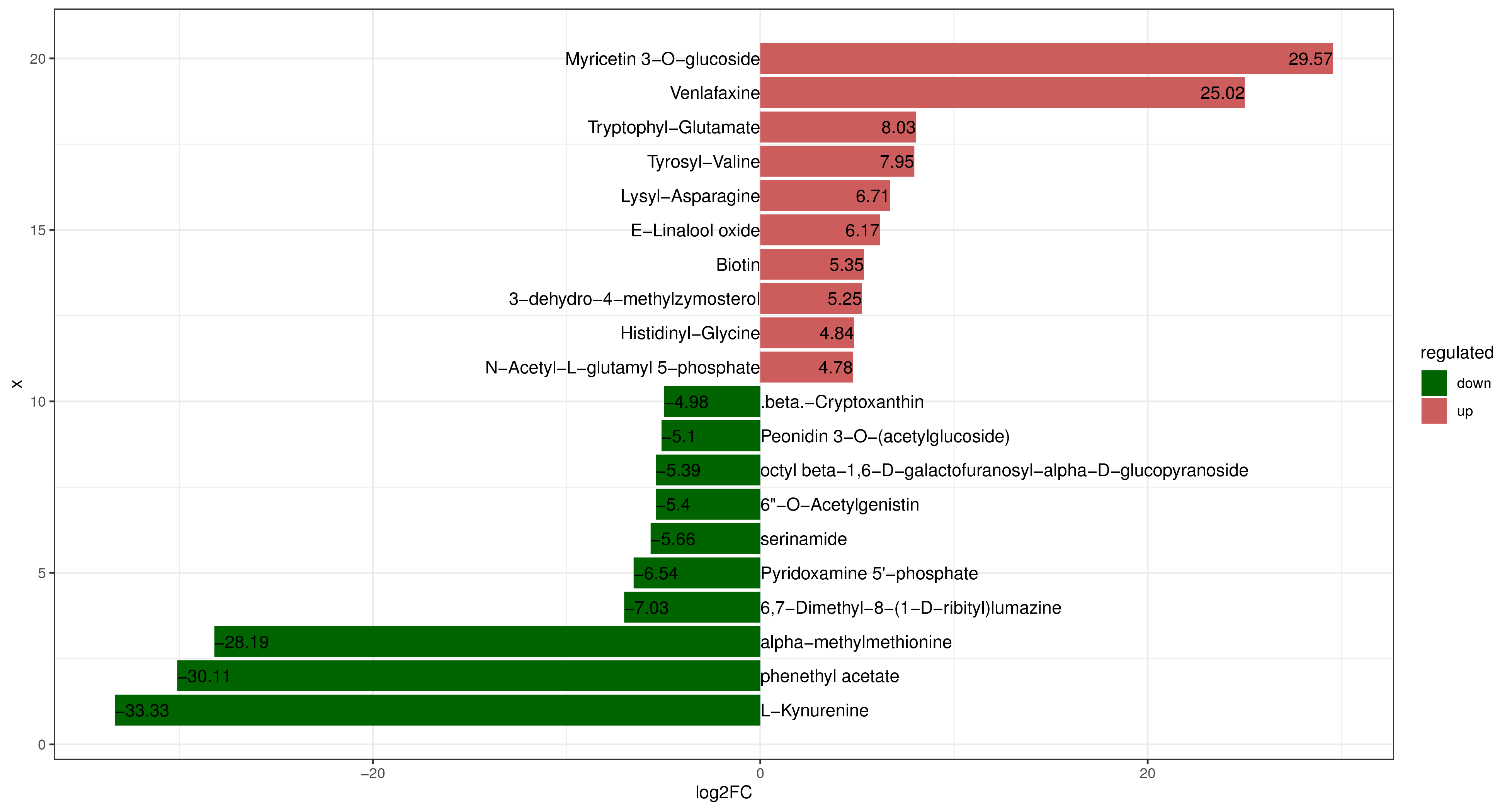


LA-MA MA-HA LA-HA

**Figure S1** Microbial metabolites in the top 10 differentially multiples were up-regulated and down-regulated

**LA-MA**


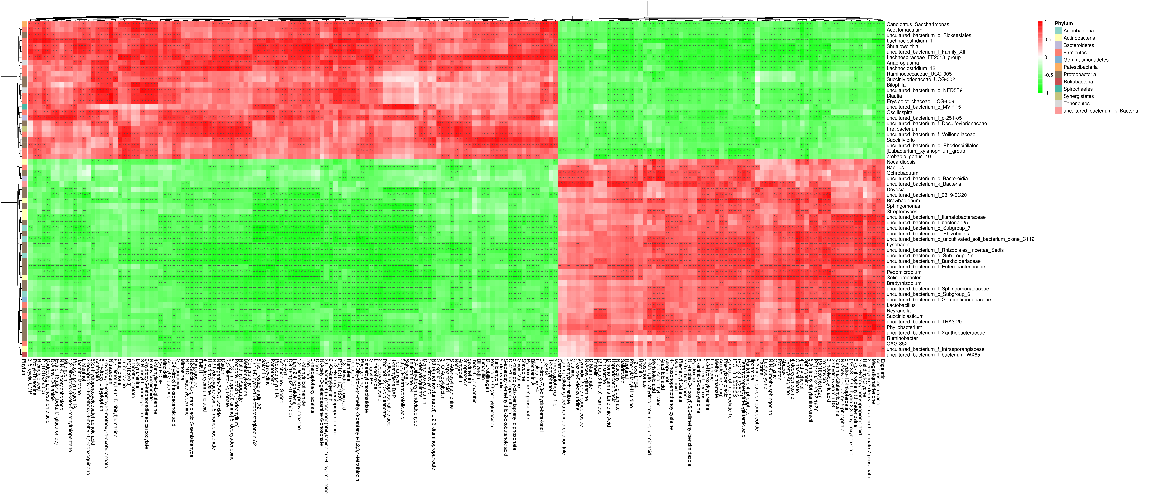


**MA-HA**


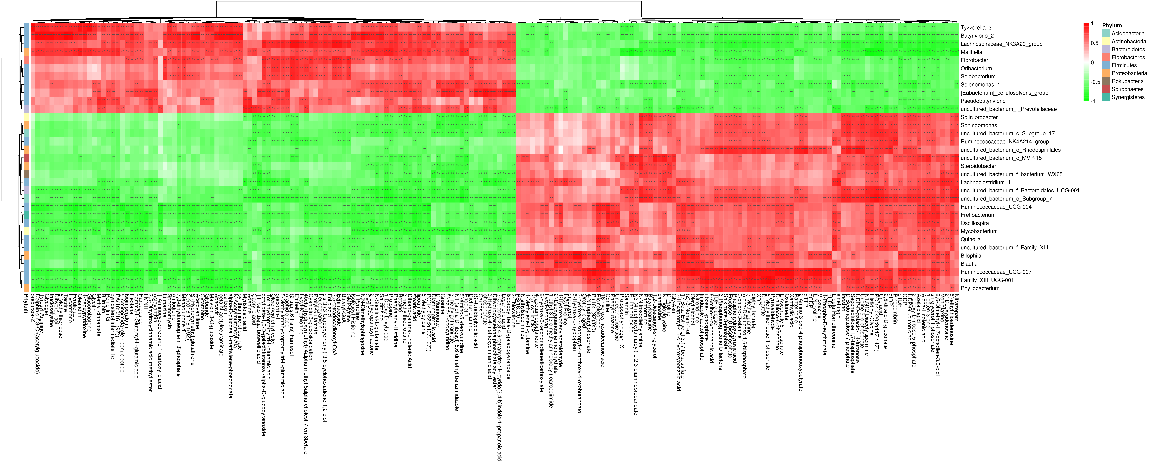


**LA-HA**


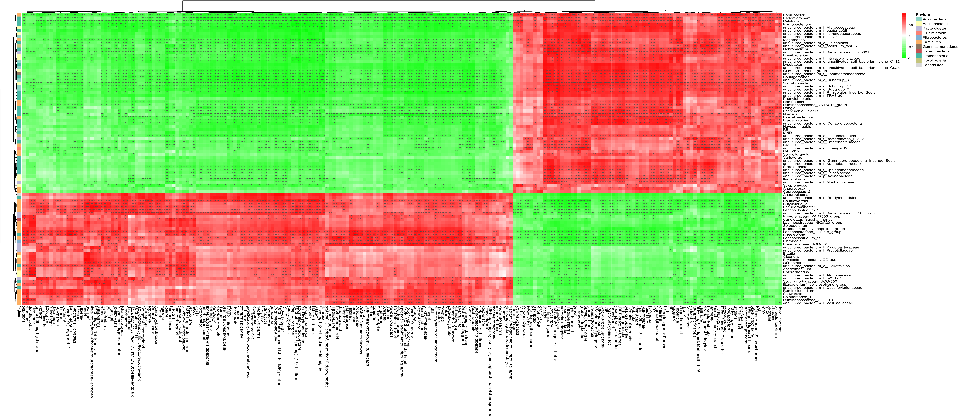


**Figure S2** Differential metabolites of microbiota - differential microbiota (generic level) correlation heat map


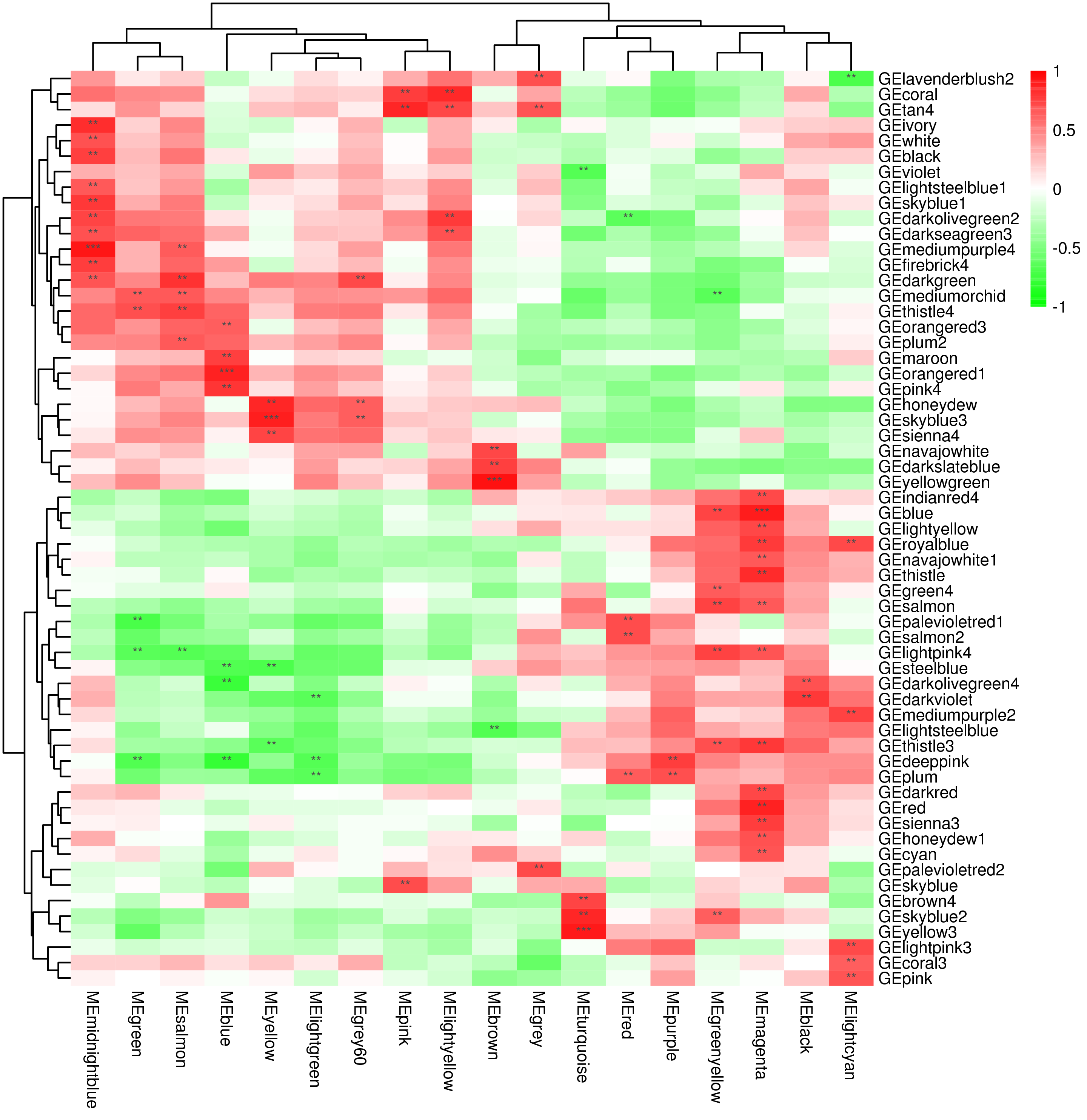

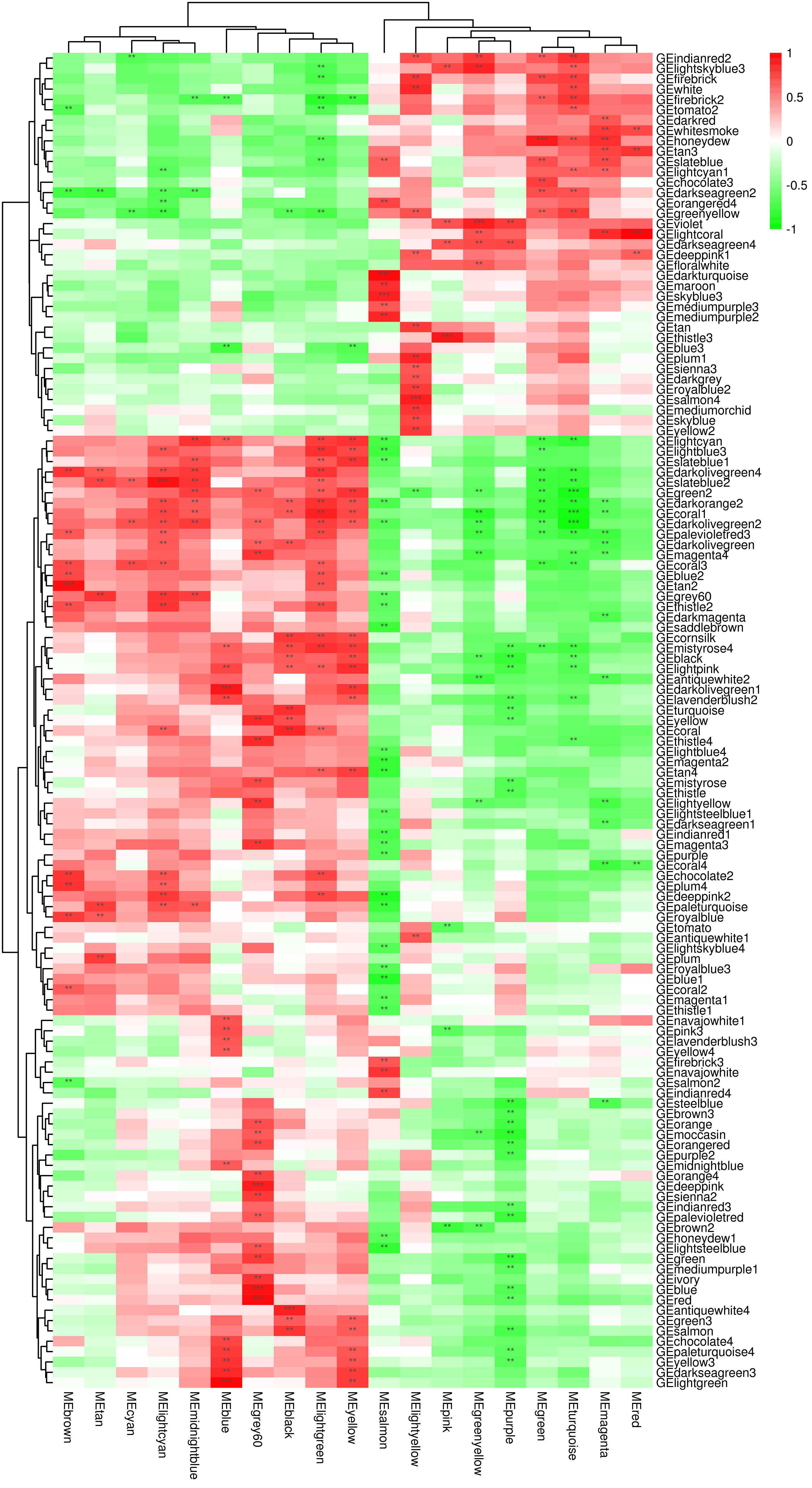

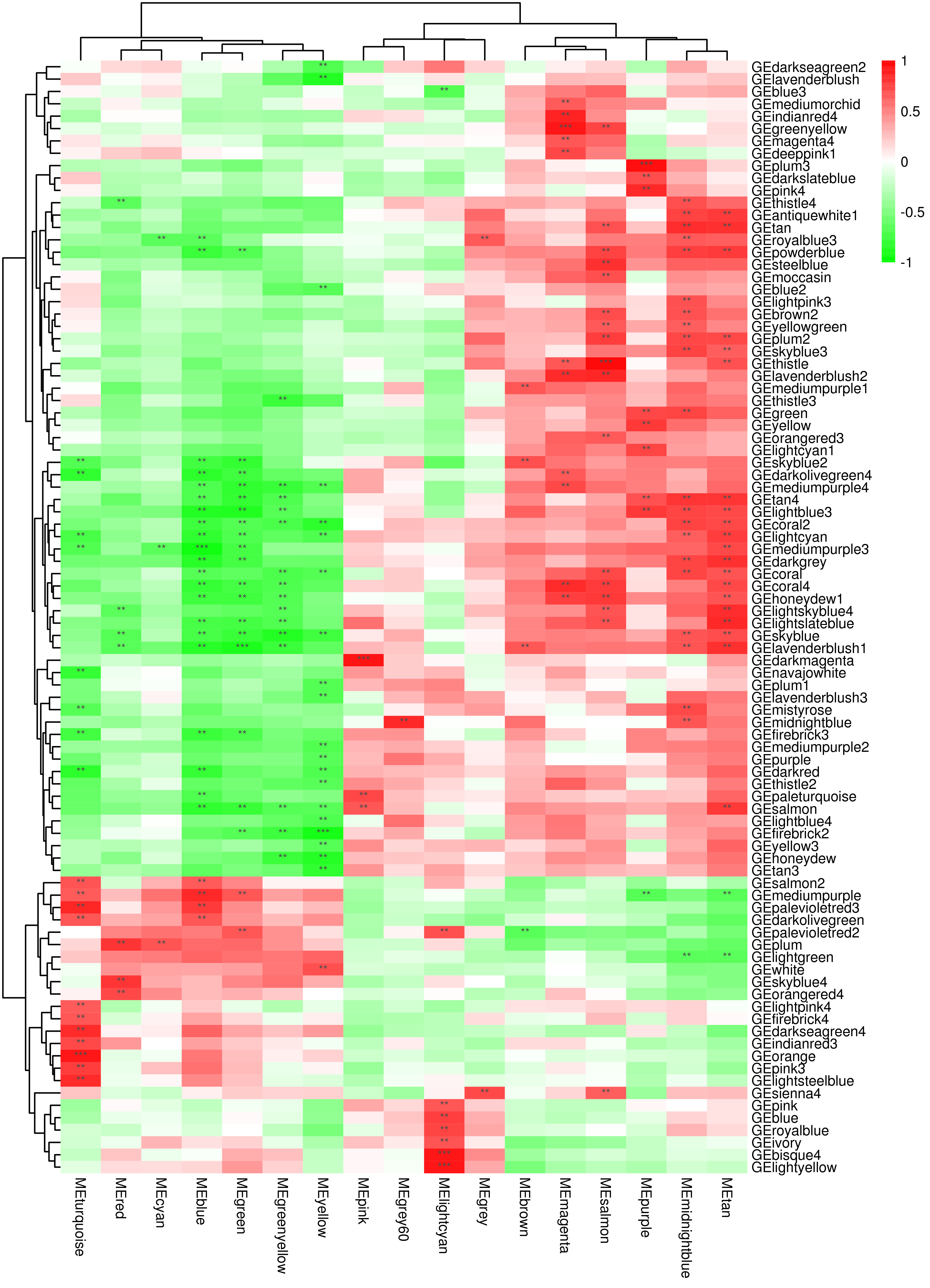


**LA-MA MA-HA LA-HA**

**Figure S3** Microbial metabolite module - gene module correlation heat map


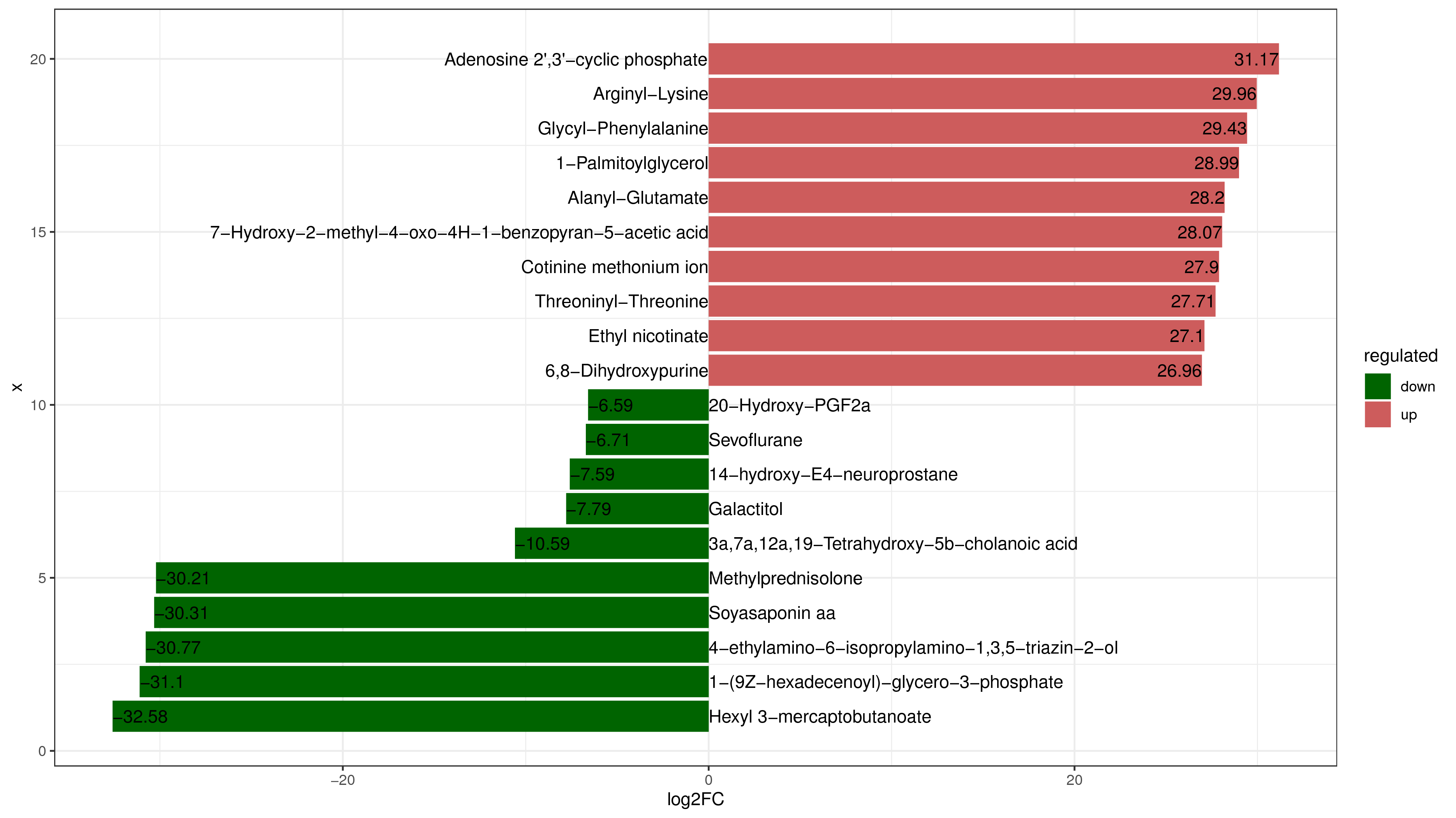

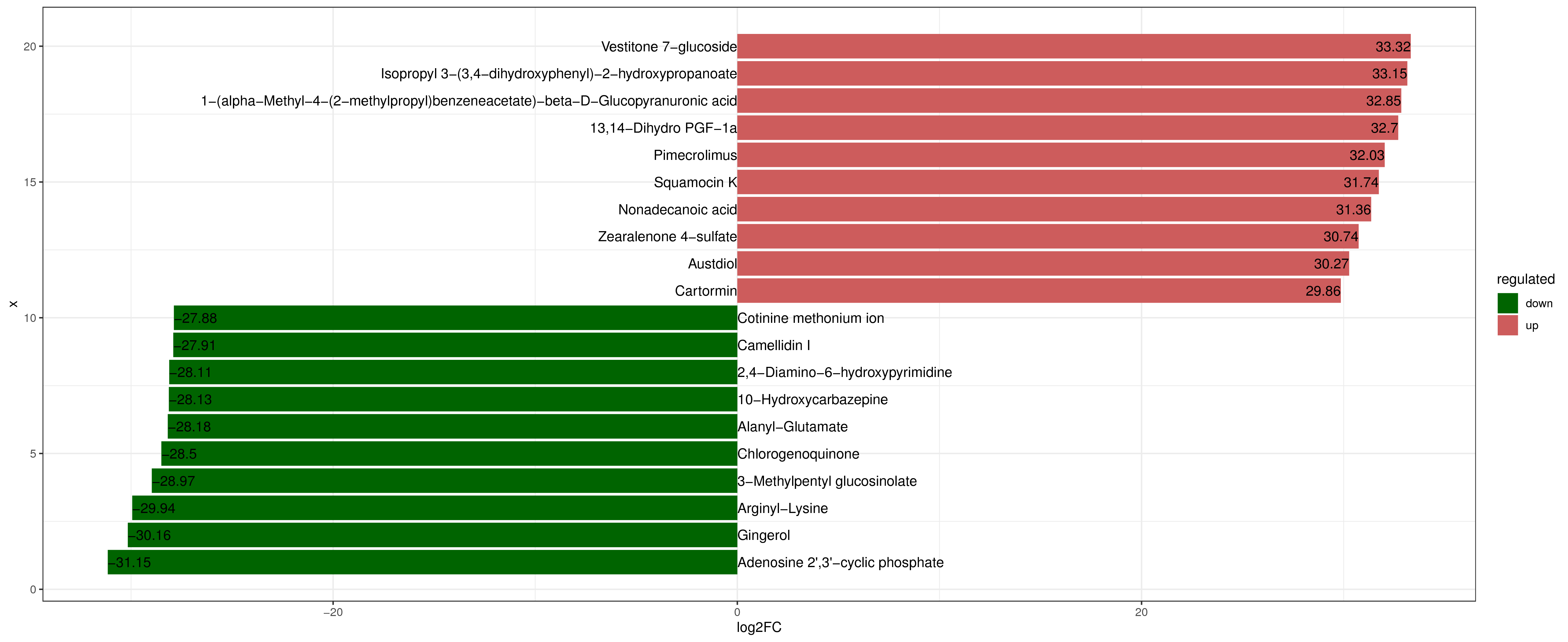

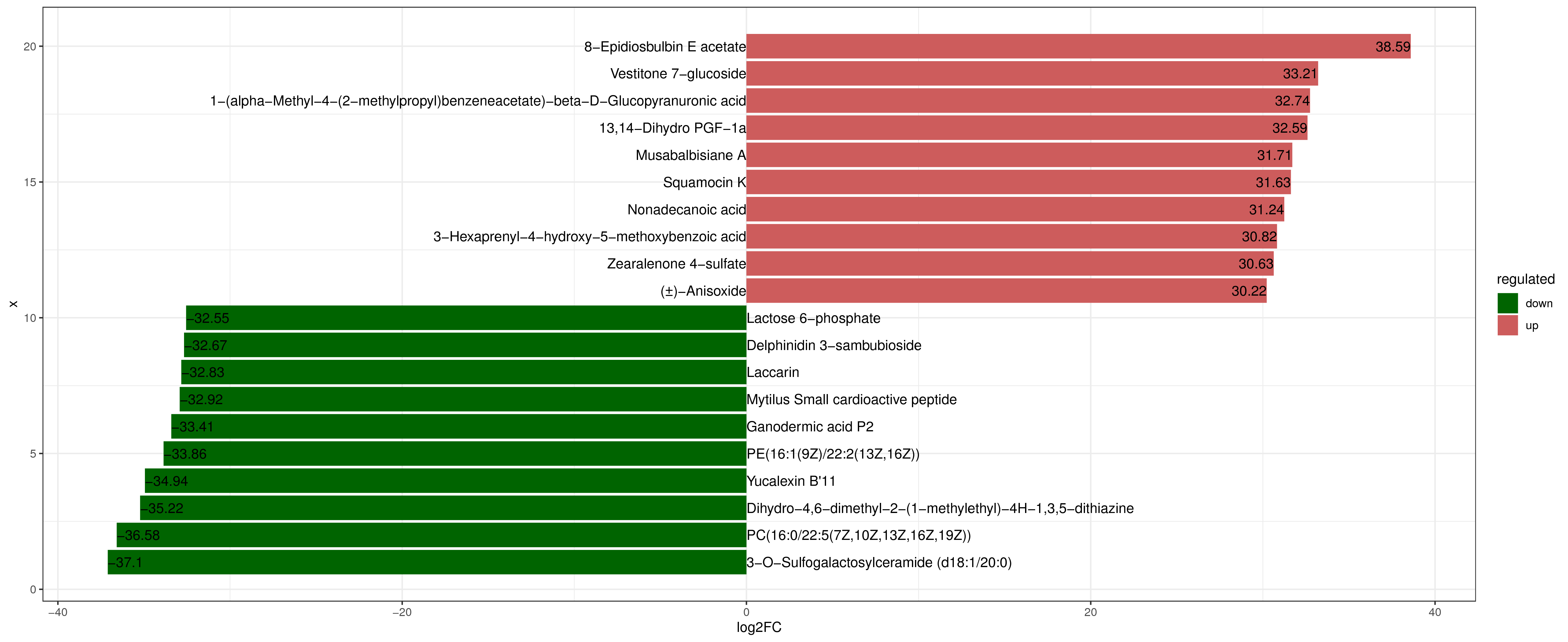


**LA-MA MA-HA LA-HA**

**Figure S4** The up and down serum metabolites of the top 10 differentially multiples

**LA-MA**


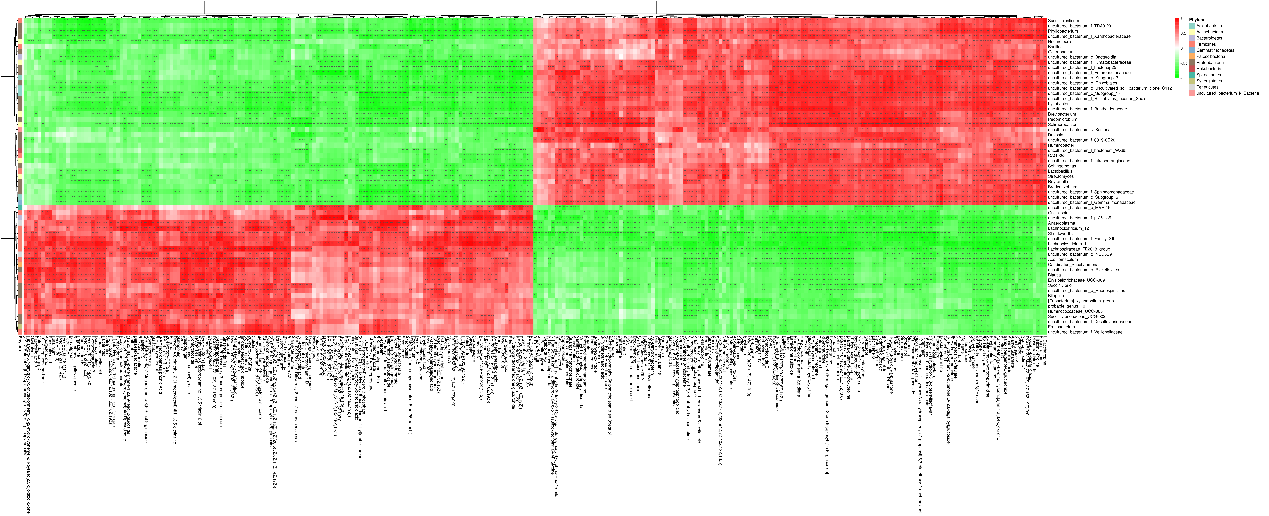


**MA-HA**


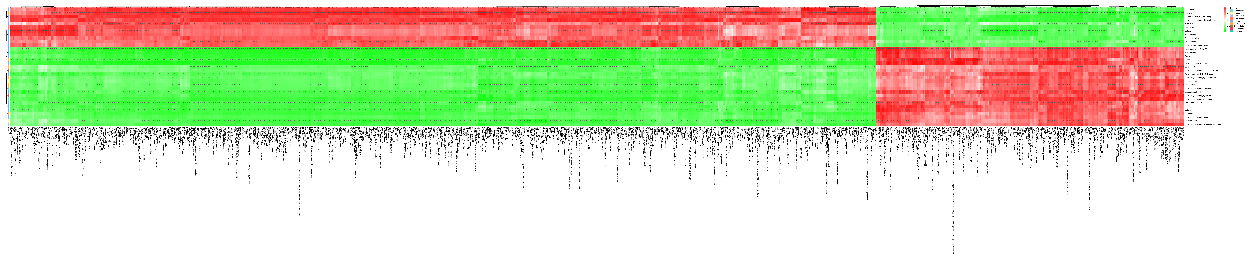


**LA-HA**


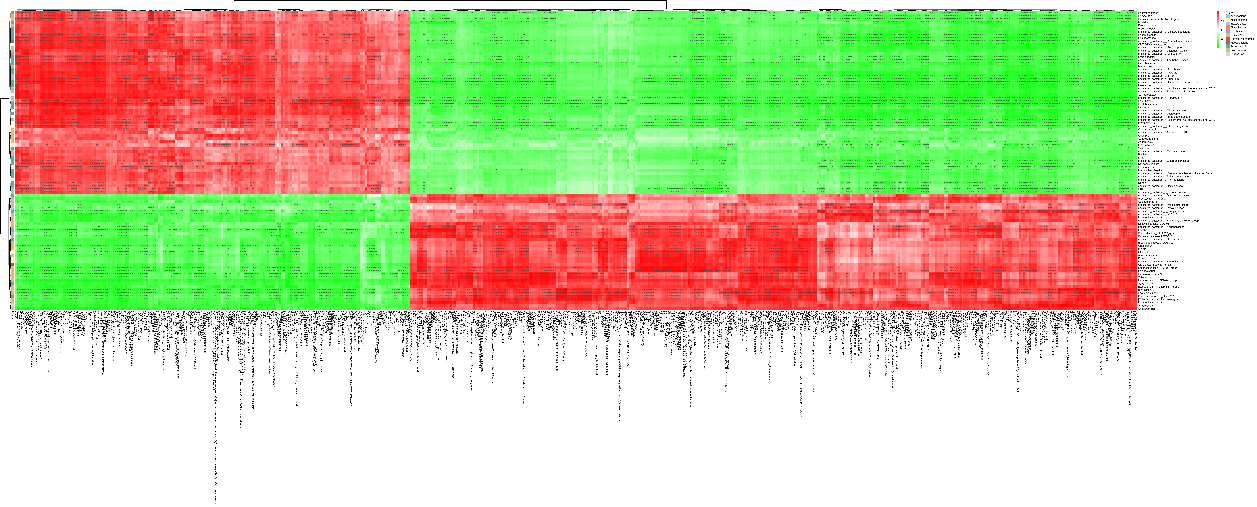


**Figure S5** Serum differential metabolites - differential microbiota (generic level) correlation heat map


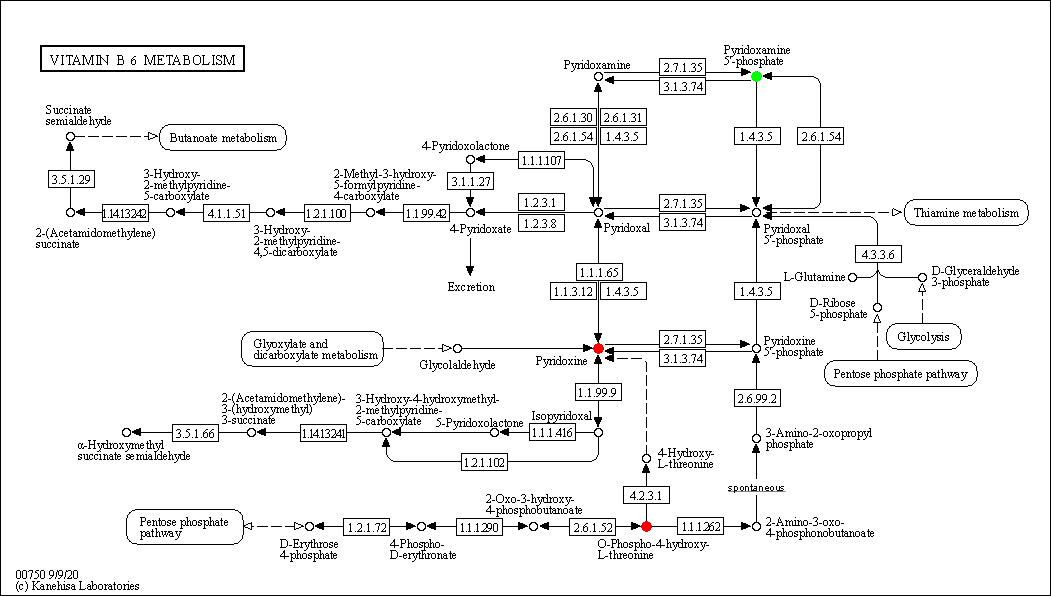


**Figure S6** Vitamin B6 metabolism

**
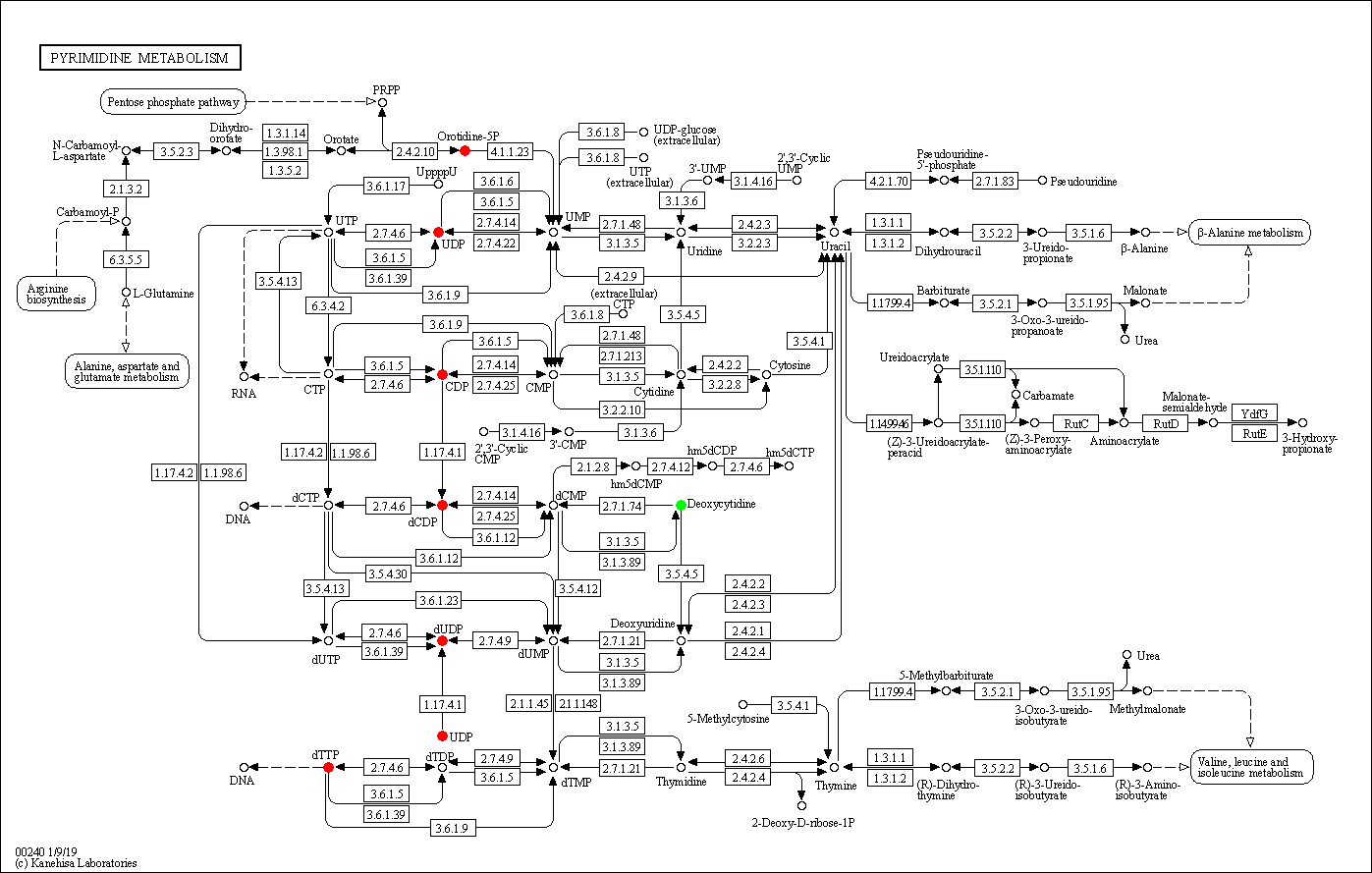
**

**Figure S7** Pyrimidine metabolism


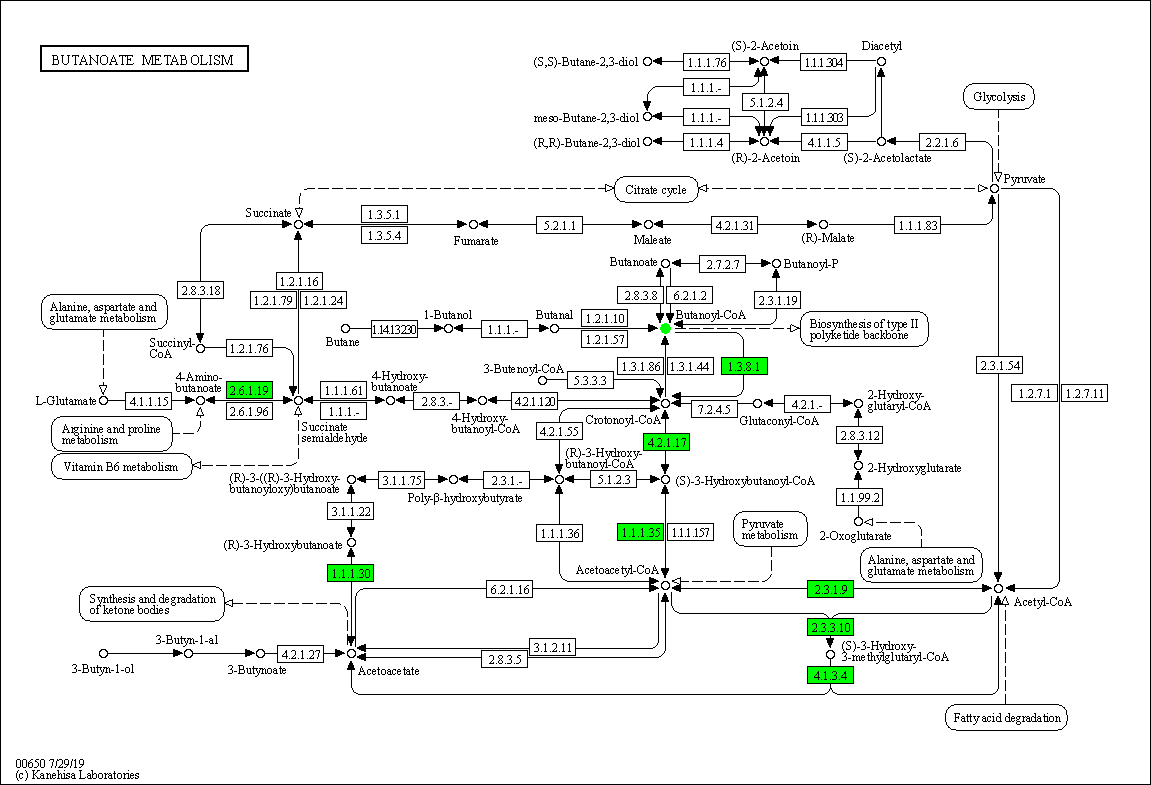


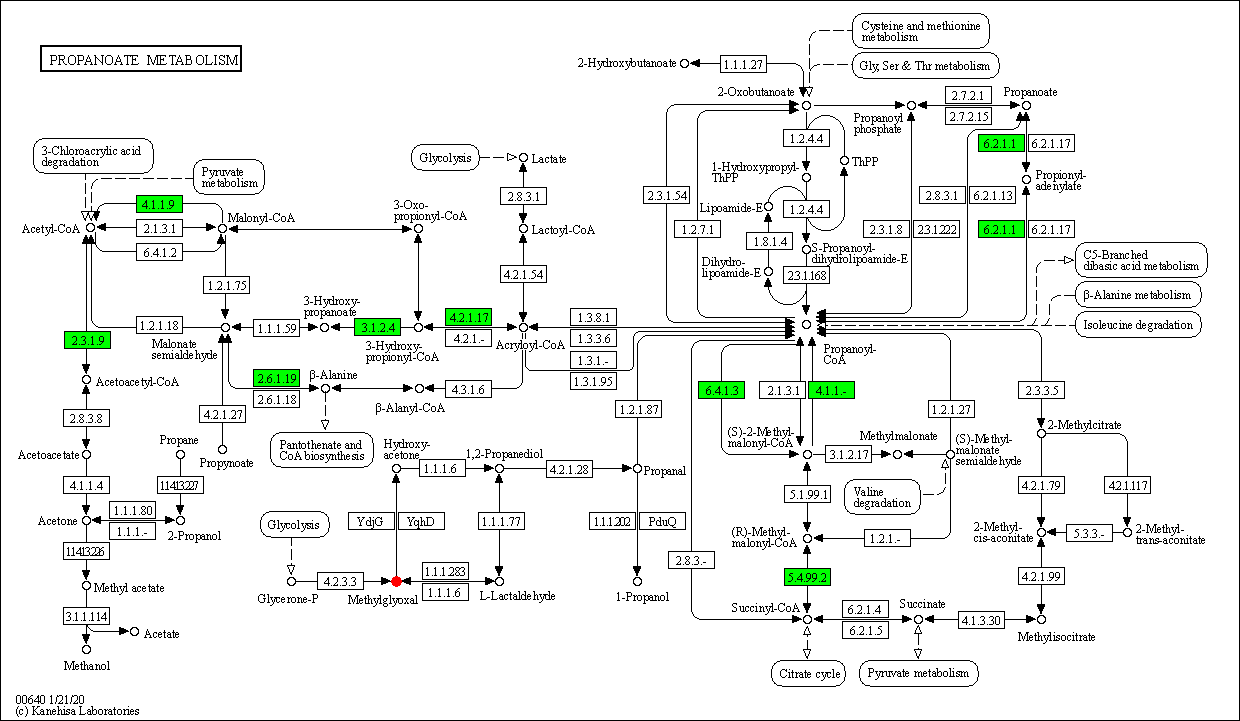


**Figure S8** Butanoate metabolism / Propanoate metabolism


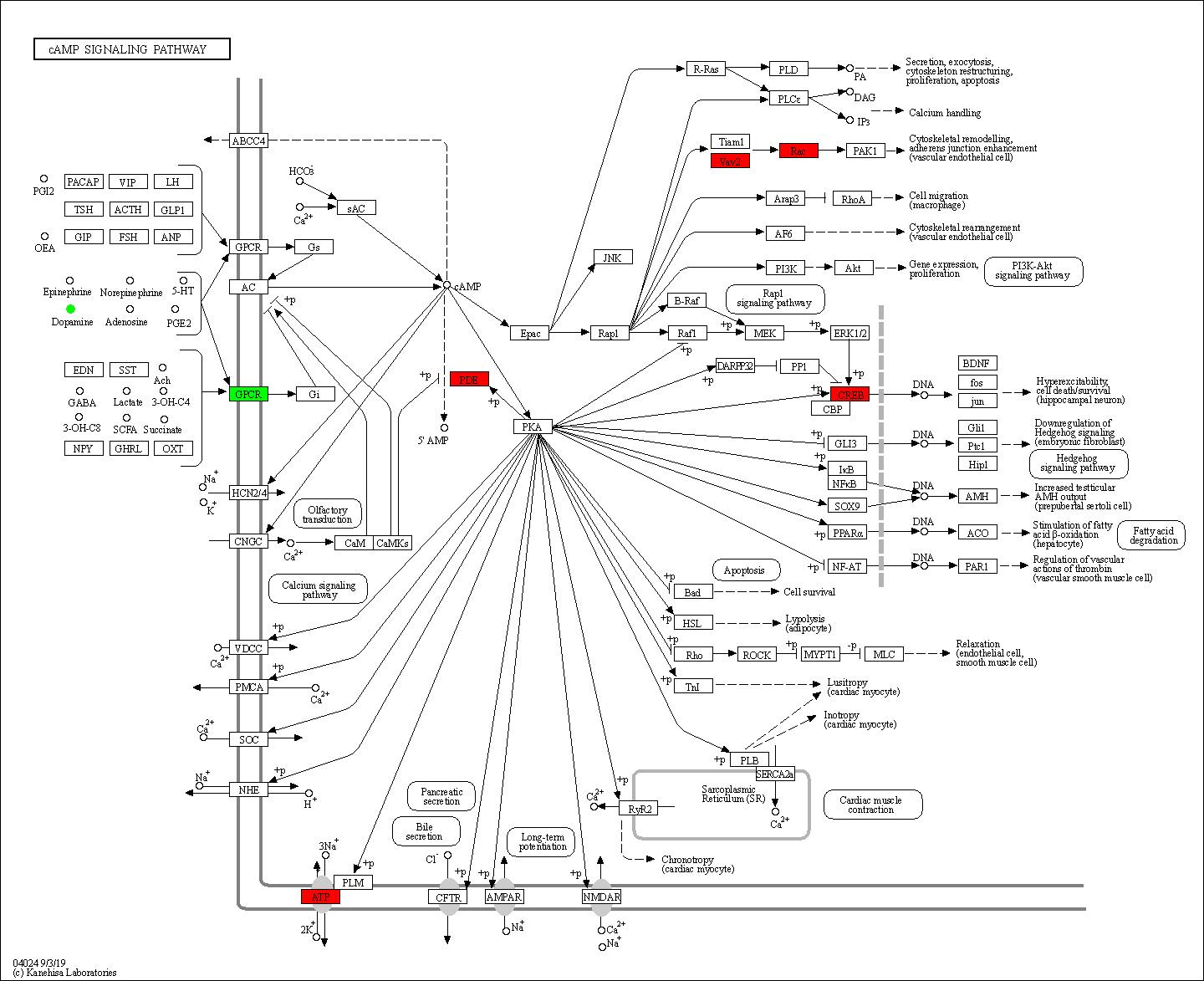


**Figure S9** cAMP signaling pathway

**Table S1** Comparative analysis of differential microbial metabolites and differential serum metabolites

| **LA-MA** | | **LA _Mean** | **MA _Mean** | **log2FC** | **Pvalue** | **regulated** |
| --- | --- | --- | --- | --- | --- | --- |
| **Metabolite** | **position** |  |  |  |  |  |
| Methyl cinnamate | Serum | 3.37×10^-5^ | 3.72×10^-4^ | 3.46 | 0.00 | up |
|  | Rumen | 4.15×10^-6^ | 8.74×10^-5^ | 4.40 | 0.003 | up |
| 8-iso-Prostaglandin A2 | Serum | 3.6×10^-5^ | 1.40×10^-6^ | -4.58 | 0.00 | down |
|  | Rumen | 8.36×10^-4^ | 3.84×10^-4^ | -1.12 | 0.00 | down |
| Geldanamycin | Serum | 2.17×10^-5^ | 1.03×10^-4^ | 2.25 | 0.00 | up |
|  | Rumen | 9.91×10^-5^ | 1.45×10^-5^ | -2.78 | 0.00 | down |

| **MA-HA** | | **MA_ Mean** | **HA_ Mean** | **log2FC** | **Pvalue** | **regulated** |
| --- | --- | --- | --- | --- | --- | --- |
| **Metabolite** | **position** |  |  |  |  |  |
| Coniine | Serum | 4.71×10^-5^ | 1.81×10^-4^ | 1.94 | 0.005 | up |
|  | Rumen | 4.80×10^-4^ | 2.74×10^-3^ | 2.51 | 0.004 | up |
| Diethanolamine | Serum | 4.50×10^-5^ | 1.39×10^-5^ | -1.69 | 0.002 | down |
|  | Rumen | 1.59×10^-4^ | 4.33×10^-5^ | -1.88 | 0.007 | down |
| Alpha-Linolenic acid | Serum | 6.84×10^-6^ | 6.88×10^-7^ | -3.31 | 0.001 | down |
|  | Rumen | 6.46×10^-4^ | 6.59×10^-3^ | 3.35 | 0.00 | up |
| 5-Hydroxy-L-tryptophan | Serum | 1.16×10^-4^ | 3.96×10^-5^ | -1.55 | 0.00 | down |
|  | Rumen | 1.71×10^-5^ | 5.50×10^-4^ | 5.01 | 0.007 | up |
| Inosine | Serum | 4.04×10^-3^ | 1.11×10^-2^ | 1.47 | 0.005 | up |
|  | Rumen | 2.19×10^-3^ | 8.59×10^-3^ | 1.98 | 0.006 | up |
| 3-Carboxy-2,3,4,9-tetrahydro-1H-pyrido[3,4-b]indole-1-propanoic acid | Serum | 1.05×10^-5^ | 1.44×10^-4^ | 3.78 | 0.005 | up |
|  | Rumen | 6.78×10^-4^ | 2.05×10^-4^ | -1.73 | 0.001 | down |
| 7-Methylguanine | Serum | 9.49×10^-6^ | 3.48×10^-5^ | 1.87 | 0.003 | up |
|  | Rumen | 3.06×10^-3^ | 6.90×10^-3^ | 1.17 | 0.001 | up |
| Lysyl-Methionine | Serum | 5.97×10^-5^ | 2.09×10^-5^ | -1.51 | 0.001 | down |
|  | Rumen | 1.43×10^-3^ | 8.99×10^-5^ | -3.99 | 0.002 | down |
| Embelin | Serum | 1.61×10^-4^ | 3.44×10^-5^ | -2.22 | 0.006 | down |
|  | Rumen | 7.27×10^-4^ | 1.59×10^-4^ | -2.20 | 0.002 | down |
| Methyl cinnamate | Serum | 3.71×10^-4^ | 2.30×10^-5^ | -4.01 | 0.00 | down |
|  | Rumen | 8.74×10^-5^ | 2.61×10^-12^ | -25.00 | 0.003 | down |

| **LA-HA** | | **LA_Mean** | **HA _Mean** | **log2FC** | **Pvalue** | **regulated** |
| --- | --- | --- | --- | --- | --- | --- |
| **Metabolite** | **position** |  |  |  |  |  |
| 3-(3-Hydroxyphenyl)propanoic acid | Serum | 1.25×10^-3^ | 2.69×10^-3^ | 1.10 | 0.007 | up |
|  | Rumen | 6.24×10^-4^ | 1.19×10^-4^ | -2.39 | 0.007 | down |
| Coniine | Serum | 1.98×10^-5^ | 1.81×10^-4^ | 3.19 | 0.003 | up |
|  | Rumen | 1.73×10^-4^ | 2.74×10^-3^ | 3.99 | 0.002 | up |
| Biochanin A | Serum | 3.10×10^-5^ | 2.61×10^-4^ | 3.07 | 0.000 | up |
|  | Rumen | 1.30×10^-4^ | 5.49×10^-5^ | -1.23 | 0.009 | down |
| Venlafaxine | Serum | 4.48×10^-6^ | 3.43×10^-7^ | -3.71 | 0.003 | down |
|  | Rumen | 2.51×10^-12^ | 8.53×10^-5^ | 25.02 | 0.009 | up |
| 2-(3-Carboxy-3-aminopropyl)-L-histidine | Serum | 1.67×10^-7^ | 3.24×10^-2^ | 17.57 | 0.005 | up |
|  | Rumen | 2.18×10^-4^ | 1.35×10^-3^ | 2.64 | 0.010 | up |
| Alpha-Linolenic acid | Serum | 1.14×10^-3^ | 3.95×10^-4^ | -1.53 | 0.001 | down |
|  | Rumen | 2.00×10^-3^ | 6.59×10^-3^ | 1.72 | 0.00 | up |
